# Interplay of Protein and DNA Structure Revealed in Simulations of the *lac* Operon

**DOI:** 10.1371/journal.pone.0056548

**Published:** 2013-02-14

**Authors:** Luke Czapla, Michael A. Grosner, David Swigon, Wilma K. Olson

**Affiliations:** 1 Department of Chemistry & Chemical Biology, Rutgers, The State University of New Jersey, Piscataway, New Jersey, United States of America; 2 BioMaPS Institute for Quantitative Biology, Rutgers, The State University of New Jersey, Piscataway, New Jersey, United States of America; 3 Department of Mathematics, University of Pittsburgh, Pittsburgh, Pennsylvania, United States of America; University of Oklahoma, United States of America

## Abstract

The *E. coli* Lac repressor is the classic textbook example of a protein that attaches to widely spaced sites along a genome and forces the intervening DNA into a loop. The short loops implicated in the regulation of the *lac* operon suggest the involvement of factors other than DNA and repressor in gene control. The molecular simulations presented here examine two likely structural contributions to the *in-vivo* looping of bacterial DNA: the distortions of the double helix introduced upon association of the highly abundant, nonspecific nucleoid protein HU and the large-scale deformations of the repressor detected in low-resolution experiments. The computations take account of the three-dimensional arrangements of nucleotides and amino acids found in crystal structures of DNA with the two proteins, the natural rest state and deformational properties of protein-free DNA, and the constraints on looping imposed by the conformation of the repressor and the orientation of bound DNA. The predicted looping propensities capture the complex, chain-length-dependent variation in repression efficacy extracted from gene expression studies and *in vitro* experiments and reveal unexpected chain-length-dependent variations in the uptake of HU, the deformation of repressor, and the folding of DNA. Both the opening of repressor and the presence of HU, at levels approximating those found *in vivo*, enhance the probability of loop formation. HU affects the global organization of the repressor and the opening of repressor influences the levels of HU binding to DNA. The length of the loop determines whether the DNA adopts antiparallel or parallel orientations on the repressor, whether the repressor is opened or closed, and how many HU molecules bind to the loop. The collective behavior of proteins and DNA is greater than the sum of the parts and hints of ways in which multiple proteins may coordinate the packaging and processing of genetic information.

## Introduction

**Figure 1:**
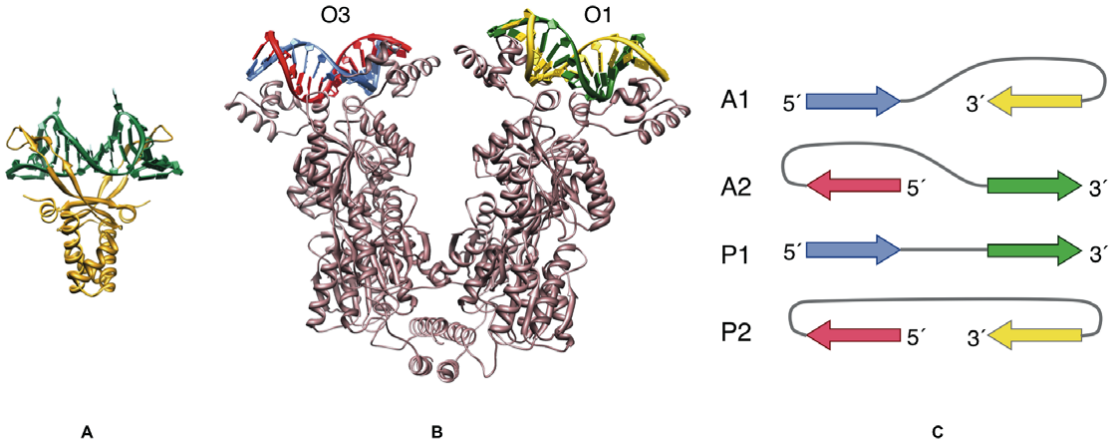
Molecular constraints imposed on protein-mediated DNA loops. (A) The non-specific architectural protein HU introduces a sharp bend in the DNA loop upon binding. (B) The Lac repressor assembly (LacR) sets the positions of the DNA operators at the ends of the loop. (C) The loop adopts one of four possible orientations on the LacR headpieces. The color-coding in B and C distinguishes the operator strands in terms of their contacts to the four LacR monomers: blue (strand A); red (strand B), yellow (strand C), green (strand D). The arrows denote the 5′-3′ directions of the operators on the binding headpieces, the characters A and P specify the antiparallel or parallel orientations of the bound operators, and the numerals 1 and 2 distinguish whether the first operator (O3) points toward the inside or outside of the assembly. The HU-bound DNA is represented by the best resolved crystal complex of the *Anabaena* protein with DNA (PDB entry 1P51) [2] and the LacR-DNA is a model obtained by composition [27] of currently available X-ray data [14], [31] (see Methods). Note the very different bending of the protein-bound DNA, toward and around HU but away from the two LacR headpieces. Molecular images rendered with Chimera [73].

The highly abundant nucleoid protein HU contributes to both the spatial organization and biological processing of bacterial DNA [1]. The dimeric protein binds with little or no sequence specificity to DNA, introducing a large bend and concomitant unwinding of the double-helical structure [2]–[5] (Figure 1A). The bending enhances the likelihood that the ends of HU-bound DNA fragments come into close contact and the chances that a short, linear molecule spontaneously adopts a tight circular arrangement [6], [7]. Indeed, the presence of HU stimulates the enzymatic closure of very short DNA chains that would otherwise remain open linear species [8], [9].

The contribution of HU to biological processing is tied to the looping of DNA induced by proteins, such as the tetrameric Lac repressor assembly (LacR), that simultaneously bind to two separate, widely spaced sites on DNA [10] (Figure 1B). The presence of HU increases the efficacy of repression of the *E. coli lactose* (*lac*) genes [11]–[13], suggesting that HU as well as LacR contribute to the looping of DNA. Deletion of the genes that code for HU drastically alters the chain-length dependent pattern of *lac* repression in modified cells [11]–[13].

The LacR tetramer is a deformable entity capable of undergoing large-scale motions away from the twofold symmetric V-shaped arrangement of protein components found in the low-resolution crystal complex with DNA [14]. Diverse experiments [15]–[22] suggest that the protein can interconvert between a tightly closed state with the arms of the “V” in intimate contact and a fully extended form with DNA binding sites located on opposite sides of the protein assembly. The mix of open and closed states varies in different studies. Whether or not such deformations play a role in gene expression remains an open question.

Current understanding of how HU and LacR might contribute to the transcription of the *lac* genes derives from indirect theoretical and computational analyses [11]–[13], [23]–[25] of the effects of DNA chain length on gene expression in *E. coli* cells with and without HU. According to these studies, which incorporate ideal elastic-rod representations of DNA in statistical mechanical treatments of the looping free energy, the DNA appears to soften in the presence of HU. The best fits of theory and computation against experiment point to substantial reduction in the effective elastic constants and concomitant changes in the apparent double-helical repeat of DNA upon HU binding. The different patterns of oscillation of gene expression with the distance between *lac* operator sites hint of a change in DNA loop type in the presence of HU [25], *i.e*., a different overall fold of DNA constrained by the precise locations of the sites on LacR to which the DNA operator sequences bind [26], [27]. Indeed, the free energies of DNA looping derived in one study [24] suggest that LacR may assemble in a fully extended, open form *in vivo*. The precise contributions of HU and LacR to DNA looping and gene expression are not clear from this perspective.

Here we take a more direct approach to the question of how proteins mediate the looping of DNA, by incorporating the known three-dimensional structural effects of both HU and LacR on DNA in Monte-Carlo simulations of the likelihood of LacR-mediated loop formation. Our work takes advantage of new methods that we have developed to study the ring-closure properties of HU-bound DNA [6], [28] and to determine the free energies of LacR-mediated DNA loops [27], [29]. We treat the double helix at the level of base-pair steps with elastic potentials that consider the intrinsic structure and deformability of successive base pairs [30], including those randomly bound by HU. We also take account of global deformations of LacR from the V-shaped structure observed in the crystalline state [14], [31] and consider the four distinct orientations of DNA operator sequences on the two LacR binding headpieces [26], [27] (Figure 1C). We determine the *J* factor of looping, an analog of the well-known measure of polymer cyclization [32], from the fraction of spatial arrangements that satisfy the restrictions on DNA end-to-end displacement and base-pair orientation imposed by the LacR assembly, and relate the computed values to the *J* factors derived from gene repression studies [11]–[13], single-molecule measurements [33]–[36], and fluorescence resonance energy transfer experiments [22].

The simulated structures immediately reveal how HU may participate in and stabilize LacR-mediated DNA looping and how HU, LacR, and DNA may work in concert to guide the chain-length-dependent variation in *lac* gene repression. The binding of HU controls the dependence of the *J* factor on chain length in ways that cannot be mimicked by an ideal elastic model of DNA. HU and DNA affect the global organization of LacR, and changes in LacR structure, in turn, alter the levels of HU binding to DNA and the orientation of DNA on LacR. The length of the DNA determines whether the operators bind in antiparallel or parallel orientations on LacR, whether the LacR is opened or closed, and how many HU molecules bind to the loop. The presence of HU limits the opening of DNA-bound LacR, increases the mix of looped states, and guides the fold of the LacR-anchored DNA. The composite interactions hint of ways in which large protein assemblies may coordinate the packaging and processing of genetic information. The unanticipated interplay of protein and DNA structure revealed in the simulations offers new ideas on how to control and enhance protein-mediated DNA looping.

## Results

### Looping of short DNA on LacR is typically easier than cyclization

**Figure 2:**
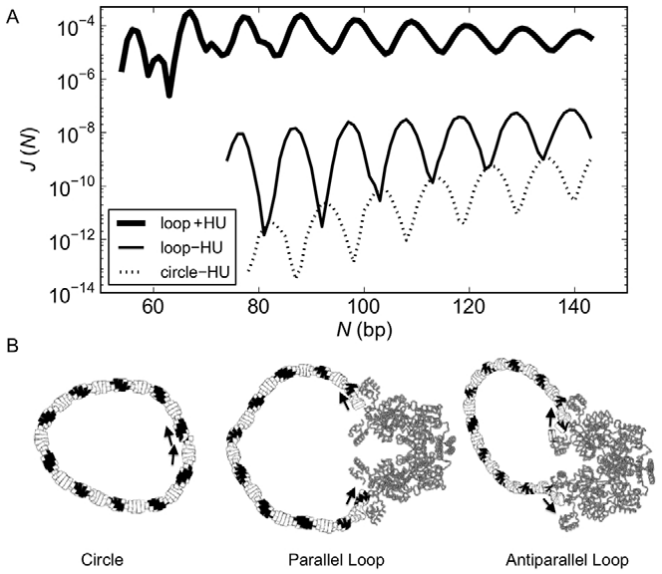
HU enhances the looping propensities of DNA. The probability of DNA looping between the headpieces of the rigid, V-shaped LacR assembly in the presence (+) or absence (−) of HU exceeds that of forming protein-free DNA minicircles of the same chain length *N*. The looping and cyclization propensities, or *J* factors [32], in (A) are obtained from the fraction of simulated configurations in ensembles of 10^12^–10^16^ fluctuating duplexes with chain ends in the requisite spatial disposition. The cartoon in (B), constructed using 3DNA [64], [65] from the base-pair step parameters of successfully closed chains, illustrates the relative deformations of DNA entailed in cyclization compared to the formation of parallel and antiparallel loops of the same chain length (105 bp) and the different constraints on DNA ends. Configurations of HU-bound loops in A are generated such that there is one HU dimer randomly bound, on average, every 150 bp of DNA in the simulated ensembles. The double helix is assumed to be naturally straight in its equilibrium rest state, inextensible, and capable of isotropic bending and independent twisting at the base-pair level. The protein-bound DNA is modeled implicitly in terms of the base-pair-step parameters found in the currently best-resolved crystal structure of operator-bound LacR [31] and the four high-resolution structures of DNA with *Anabaena* HU [2]–[4]. The shading of blocks in B denotes the minor-groove edges of the base pairs and the small arrows the end-to-end separation and direction of terminal base pairs. Note that the illustrated loops include only the (inner) halves of the bound operators counted in the chain length.

Short DNA chains close more easily into LacR-mediated loops than form minicircles at most chain lengths (Figure 2A). Cyclization imposes more restrictive constraints on the spatial pathways of DNA than looping. The last base pair of a successfully closed linear duplex must not only approach the first base pair but also make that approach from behind, with the chain leaving and returning to its origin in the same direction (Figure 2B). By contrast, the ends of a DNA fragment looped between the binding sites of a protein lie farther apart and the intervening residues can leave and return to the anchoring sites in the same or the opposite direction. In both types of structures the ends of the chain must satisfy orientational constraints that allow for either chemical bond formation or specific interactions with protein (see Methods). These requirements are best met if the ends of the chain are appropriately phased with the ∼10.5 bp DNA helical repeat. Chains differing in length by 5–6 base pairs (bp) thus exhibit very different propensities to adopt a constrained configuration.

Here we compare the ease of looping a DNA fragment of chain length *N* between the binding headpieces of the Lac repressor assembly and the cyclization of a DNA molecule of the same length. We define the length of the DNA loops in the conventional sense, as the number of base pairs between the centers of the bound operator sequences. The loops thus include seven rigid base pairs from the inner half of each operator and *N* – 14 bp of deformable DNA. All *N* bp of the minicircle undergo the same types of deformations as those imposed on the protein-free segments of the loops. The rigid base pairs on the bound operators follow the trajectory of DNA observed in the high-resolution crystal complex of a LacR dimer with a symmetric operator [31] and the deformable DNA is subject to standard fluctuations in bending and twisting about the canonical B-type structure [6], [28] (see Methods). The HU bound to DNA adopts one of the pathways found in the crystal complexes with the *Anabaena* protein [2].

The very different constraints on the ends of the minicircles and loops give rise to a phase shift in the chain-length dependent variation in the *J* factors of the two types of structures (Figure 2A) and also in loops of different types (Figure S1 in the Supporting Information). The offset in maxima and minima thus leads to striking differences in the ease of looping compared to cyclization at chain lengths where looping is highly probable and cyclization is less likely, *i.e*., values of *N* where there is a local peak in the computed *J* factors of looping and a dip in the *J* factors of cyclization. The probability of forming a DNA loop bound to the recognition headpieces of a rigid, V-shaped LacR structure in the absence of HU, *i.e*., the ease of introducing a protein-free DNA segment between the LacR-bound nucleotides in the model in Figure 1B, can be 4–5 orders of magnitude greater than that associated with the covalent closure of DNA of the same chain length and an additional 3–5 orders of magnitude more likely if formed on the same rigid assembly in the presence of HU (at levels of one randomly bound HU dimer for every 150 bp of DNA). The likelihood of chain cyclization in the absence of the structural protein, however, slightly exceeds that of looping under the same conditions at a few chain lengths where the DNA is less likely to adopt the end-to-end arrangements required for binding to the rigid V-shaped LacR complex. By contrast, the probability of loop formation in the presence of HU always exceeds that of circularization in the absence of the protein.

### HU modulates the chain-length dependence of LacR-mediated looping

The introduction of HU perturbs the regular oscillations in the *J* factor with chain length found for both ring closure and looping in the absence of the architectural protein (Figure 2). In contrast to ring closure and looping without HU, where the chances of the chain ends coming into appropriate contact increase with chain length between 75 and 150 bp, the probability of LacR-mediated DNA loop formation in the presence of HU is roughly comparable in short chains of lengths differing by multiples of a helical turn (10–11 bp). That is, the magnitudes of the peaks and valleys in the *J* factors of looping show little, if any, dependence on chain length over the range of values considered here. The bound HU dampens the oscillations in *J* so that LacR-mediated loops are easily formed at most chain lengths. The peaks and valleys in the latter looping profile are 5–6 bp out of phase from those associated with cyclization in the presence of HU [6]. The different constraints imposed on the ends of the successfully closed loops compared to those placed on a circular structure of the same chain length, in combination with the local deformations of DNA induced by HU, give rise to the shifts in phase and magnitude of the simulated curves.

### HU increases the mix of DNA loops formed on V-shaped LacR

**Figure 3:**
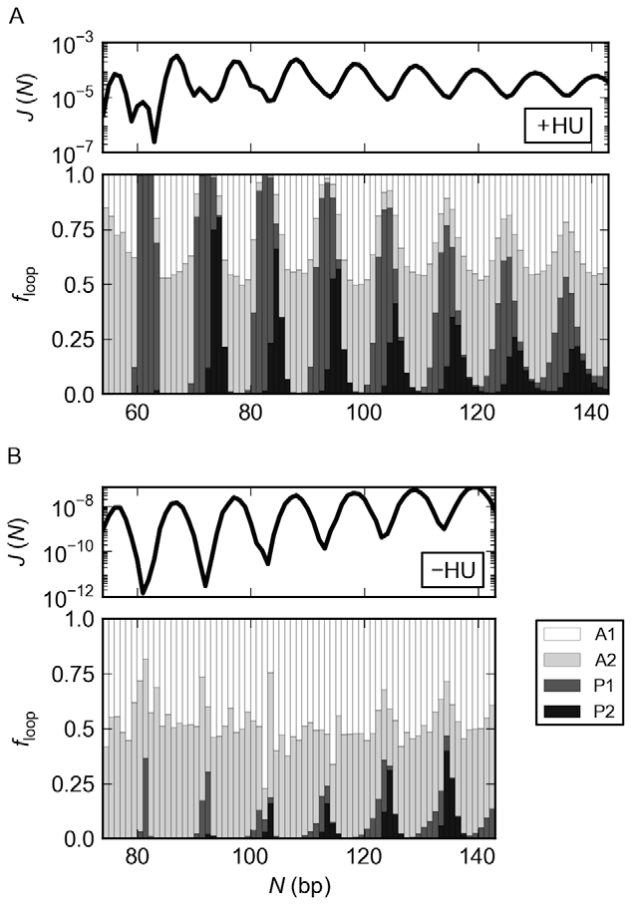
HU increases the mix of DNA loops formed on LacR. The types of DNA loops formed on the V-shaped LacR assembly underlie the chain-length-dependent variation in the *J* factor. Note the more complex plot of *J*(*N*) and the greater diversity of loops obtained in the presence (A) compared to the absence (B) of randomly bound HU molecules. The diversity is expressed in terms of the fraction of loops *f*_loop_ with DNA bound to LacR in one of the four specified orientations. See Figure 1C for schematics of the antiparallel (A1, A2) and parallel (P1, P2) forms and the legend to Figure 2 for computational details. The wider range of *N* in (A) *vs*. (B) reflects the greater ease of forming short LacR-mediated loops in the presence of HU.

The complex chain-length-dependent variation of *J* with chain length in the HU-bound loops reflects the predominance of different types of DNA loops at different chain lengths, *i.e*., different orientations of bound DNA on the protein assembly (Figure 1C). The loops responsible for the higher peaks in the plot of *J vs*. *N* bind to the LacR headpieces in an antiparallel fashion and those associated with the secondary peaks in a parallel fashion (compare the populations of the antiparallel (A1, A2) and parallel (P1, P2) loops with *N* and the corresponding plot of *J vs*. *N* in Figure 3A). The secondary peaks in *J* become less apparent at longer chain lengths, where they contribute to broad, asymmetric valleys in the *J*(*N*) profile. Although some very short HU-decorated loops adopt exclusively parallel or antiparallel orientations, most DNA fragments bind to the rigid LacR headpieces in either orientation. The likelihood of DNA looping in both orientations on LacR increases with increase in chain length. The oscillatory changes in loop type stem from the different spatial constraints imposed on the ends of parallel vs. antiparallel loops (see Figure 2B).

The DNA loops bound to the rigid LacR assembly in the absence of HU show a less pronounced, chain-length-dependent alternation of loop type (Figure 3B). The computed preference for antiparallel looping at different chain lengths agrees with our earlier analytical assessment of the cost of looping DNA in all possible orientations on the rigid V-shaped LacR assembly [27], [29]. The small fraction of parallel loops found here occurs at chain lengths in the vicinity of the local dips in the *J* factor. Close examination of the *J*(*N*) plot (Figure 3B) shows that these states occur at values of *N* where there is a slight shoulder in the curve brought about by the secondary looped configurations. The *J* factors associated with the formation of the four kinds of LacR-mediated loops reveal the greater difficulty in binding DNA in parallel compared to antiparallel orientations on the protein assembly (see Figure S1 in the Supporting Information).

### DNA chain length controls the uptake of HU on LacR-mediated loops

**Figure 4:**
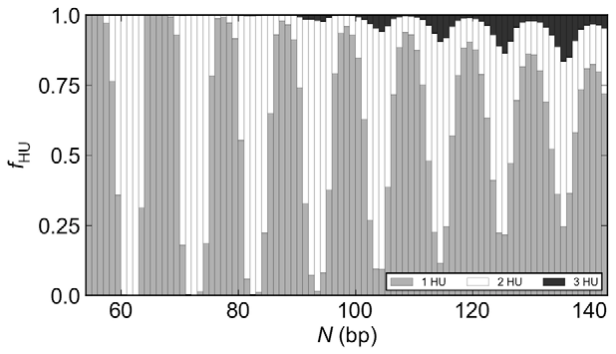
DNA chain length controls the uptake of HU on LacR-mediated loops. The distribution of HU molecules bound to DNA loops mediated by the rigid, V-shaped LacR protein assembly varies regularly and abruptly with chain length *N*. The value of *f*_HU_ corresponds to the fraction of loops with the specified number of bound HU dimers. See the legend to Figure 2.

In addition to the variation in binding orientation, the LacR-mediated DNA loops formed on the rigid V-shaped assembly in the presence of HU take up different numbers of proteins at different chain lengths (Figure 4). The amount of bound HU depends upon loop type. The antiparallel loops that contribute to the local maxima in the *J* factors typically bind a single HU dimer, whereas the parallel loops found at the dips in *J* generally take up 2–3 dimers. Moreover, the HU composition in the antiparallel loops changes abruptly with increase in chain length, particularly at low values of *N*. The parallel loops, by contrast, tend to bind two HU dimers regardless of chain length. Small populations of parallel loops with one or three bound HU dimers accumulate with increase of chain length. See Figure S2 in the Supporting Information for the chain-length-dependent variation in the number of HU dimers bound to the four types of DNA loops formed on the rigid LacR complex.

### DNA looping on rigid LacR mimics key *in-vivo* looping properties

**Figure 5:**
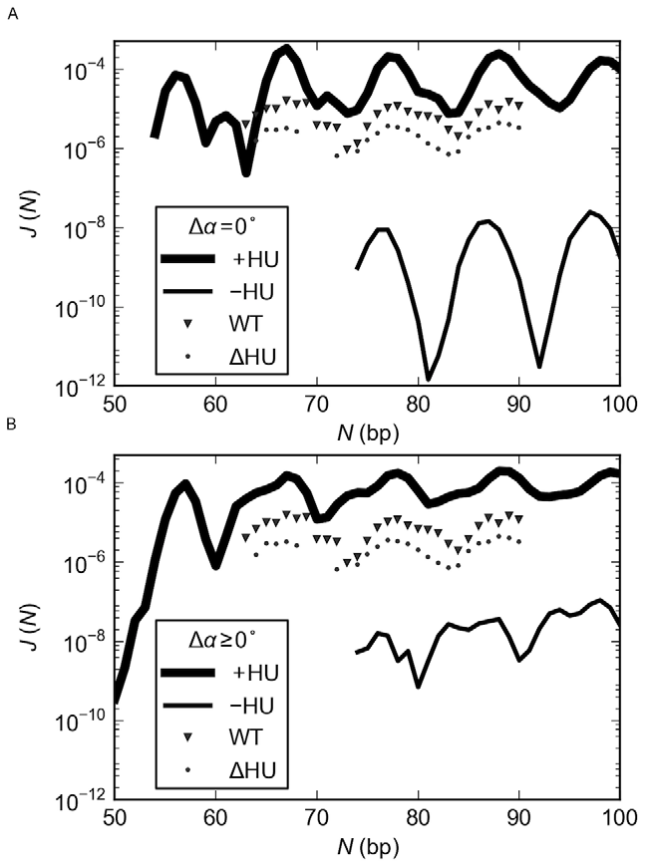
DNA loops simulated in the presence of HU and deformable LacR capture *in-vivo* looping properties. The predicted ease of DNA loop formation between the headpieces of (A) a rigid V-shaped LacR complex and (B) a deformable LacR assembly mimics looping propensities deduced from the *in-vivo* expression of *lac* genes [11]–[13]. The chain-length-dependent gene-expression profiles of *E. coli* cells are expressed as *J* factors using Eqn. (4). Values of *J* for a strain with wild-type (WT) proteins and a mutated strain (ΔHU) that cannot express HU are depicted respectively by large and small dark symbols, with data reported in different publications for the same DNA construct [11]–[13] expressed as average values. The simulated *J* factors reflect the likelihood that an ideal, naturally straight DNA molecule folds along a pathway compatible with the spatial constraints imposed by the binding of LacR and the presence or absence of HU (points connected respectively by thick and thin lines).

The *J* factors of DNA loops attached to the rigid LacR assembly (Figure 5A) mimic the complex, chain-length-dependent variation in looping propensities deduced from gene-expression profiles [11]–[13], here re-expressed as *J* factors (see Methods). Specifically, the simulations performed in the presence of HU capture the peaks and valleys in looping occurrences detected in wild-type cells and successfully distinguish the primary and secondary peaks in these data. The computed values of the *J* factor and the amplitudes of the *J*(*N*) profiles of the HU-bound loops, however, exceed those extracted from experiment. The simulations performed in the absence of HU show the decrease in gene-expression levels found in mutant cells that do not express HU [12], [13] as well as the observed oscillatory variation in *J* with chain length. The computed looping probabilities, however, fall substantially below those found upon deletion of the HU gene, and the amplitude of the simulated *J*(*N*) profile of HU-free DNA greatly exceeds that deduced from experiment.

### Opening of LacR captures other known looping properties

Allowance for global deformations of the LacR assembly dampens the simulated variation in *J* with chain length compared to that found under identical conditions with the rigid LacR template (Figure 5B). The model assumed here in the absence of high-resolution structural information — the ‘free’ (energetically unpenalized) opening of LacR between the V-shaped crystallographic structure and a fully extended form via rotational changes Δ*α* about an axis through the four-helix bundle that holds the two arms of the complex in place (see Methods) — significantly enhances the looping of DNA fragments of the lengths least likely to form on the rigid LacR template, the reference state where Δ*α* is zero. The deep local minima in the *J*(*N*) profiles of HU-free DNA in Figure 5A become secondary maxima or shoulders on primary maxima in Figure 5B. In addition, some of the higher peaks in *J* occur at slightly longer chain lengths than those associated with the rigid LacR model. These changes in phase reflect the changes in twist imposed on the ends of the DNA loop by the simple opening motion. Alternate pathways that couple rotations about the long axes of the LacR arms with global opening of the complex and/or impose an energy penalty on large-scale deformation [27] could preserve this phasing. On the other hand, the amplitudes of the *J*(*N*) profiles determined using the deformable LacR model compare more favorably with the range of values extracted from *lac*-expression studies than those found for loops formed on the rigid LacR structure. Moreover, the deviations between the predicted looping propensities of the HU-free DNA attached to the deformable repressor protein and the repression levels found in mutant cells are smaller than the corresponding differences obtained with the V-shaped model. The variation in LacR has a lesser effect on the *J* factors of the HU-bound loops, *i.e*., the deviations between predicted and observed values are comparable for loops formed on rigid and deformable LacR. As described in further detail below, the structural response of DNA to the assumed opening of the LacR assembly differs in the presence or absence of HU.

### DNA loop length and HU levels guide the opening of LacR

**Figure 6:**
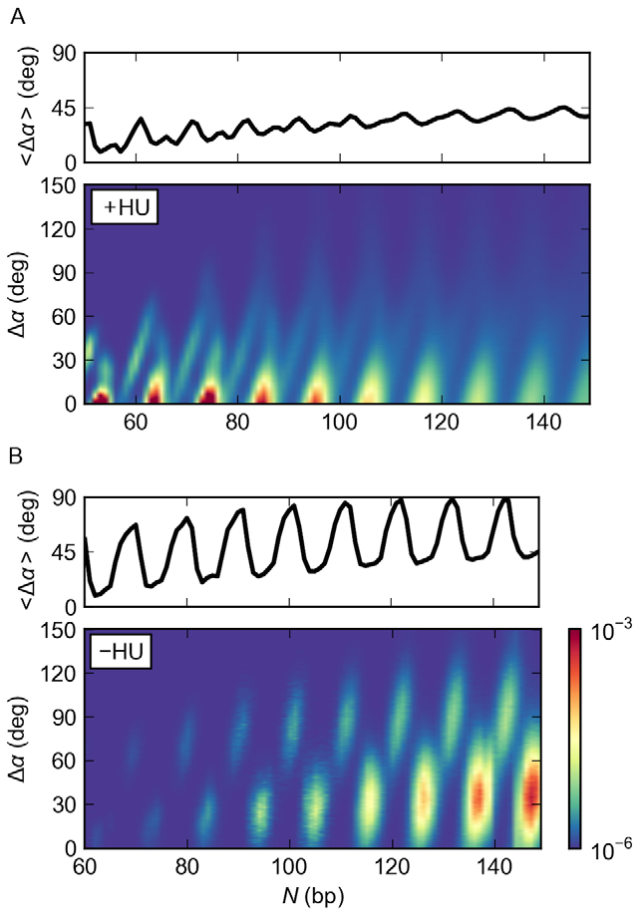
DNA loop length and HU levels govern the opening of LacR. Contour surfaces of the likelihood of DNA loop formation as a function of chain length *N* and the change in the LacR opening angle Δ*α* reveal the deformations of the repressor induced by DNA chain length and suppressed by HU. Note the narrower range and lesser variation in Δ*α* found in loops (A) simulated in the presence of randomly bound HU compared to (B) those generated in the absence of the architectural protein. The average values of the opening angle at each chain length are reported in the line plots above the contour surfaces. The blue-to-red scale on the lower right denotes the probability of loop formation over the specified range of opening angles and chain lengths. Red denotes the more easily formed loops with higher *J* factors and blue the loops with lower *J* factors.

The changes in the *J* factors associated with the opening of LacR reflect unexpected changes in the configurations of the DNA loops that are formed between the binding headpieces of the tetrameric assembly. The separation of the bound operators, *i.e*., the distance between the ends of the intervening loop, increases with increase in the angle between the arms of LacR. The degree of opening in the complex assembly depends upon both the DNA chain length and the presence or absence of HU (Figure 6). For example, the change in the angle of opening, Δ*α*, between the arms of the LacR assembly is less pronounced when bound to DNA of lengths more likely to close into loops (points of higher density on the contour plots), and the variation in the opening angle with chain length is muted in the presence compared to the absence of HU.

In the absence of HU, the opening angle exhibits a strong periodic dependence on chain length (Figure 6B). The LacR assembly opens by 10–40° from the V-shaped structure when bound to the ends of a DNA loop corresponding to one of the higher peaks in the *J* factor and by 60–120° when associated with a loop where there is a secondary peak in *J*. The full range of LacR opening occurs for HU-free DNA loops at selected intermediate chain lengths, *e.g*., loops with center-to-center inter-operator distances of 113, 123, 134, or 144 bp. By contrast, the opening of the LacR proteins that anchor DNA loops in the presence of HU is limited (Figure 6A). The LacR opening angle is 10–15° on average when attached to loops with a *J* factor at or near a local maximum and 30–40° when bound to loops with a *J* factor in the vicinity of a local minimum. The mean angle between the protein arms, however, slowly increases with the length of bound DNA (see line plots above the respective contour surfaces in Figure 6). As a result, roughly 20% of the LacR tetramers that incorporate HU-bound DNA loops of 140–150 bp open by 50° or more, and the majority of repressors attached to HU-free DNA of these lengths deform beyond this extent.

### LacR deformability changes the mix of DNA loops and levels of bound HU

**Figure 7:**
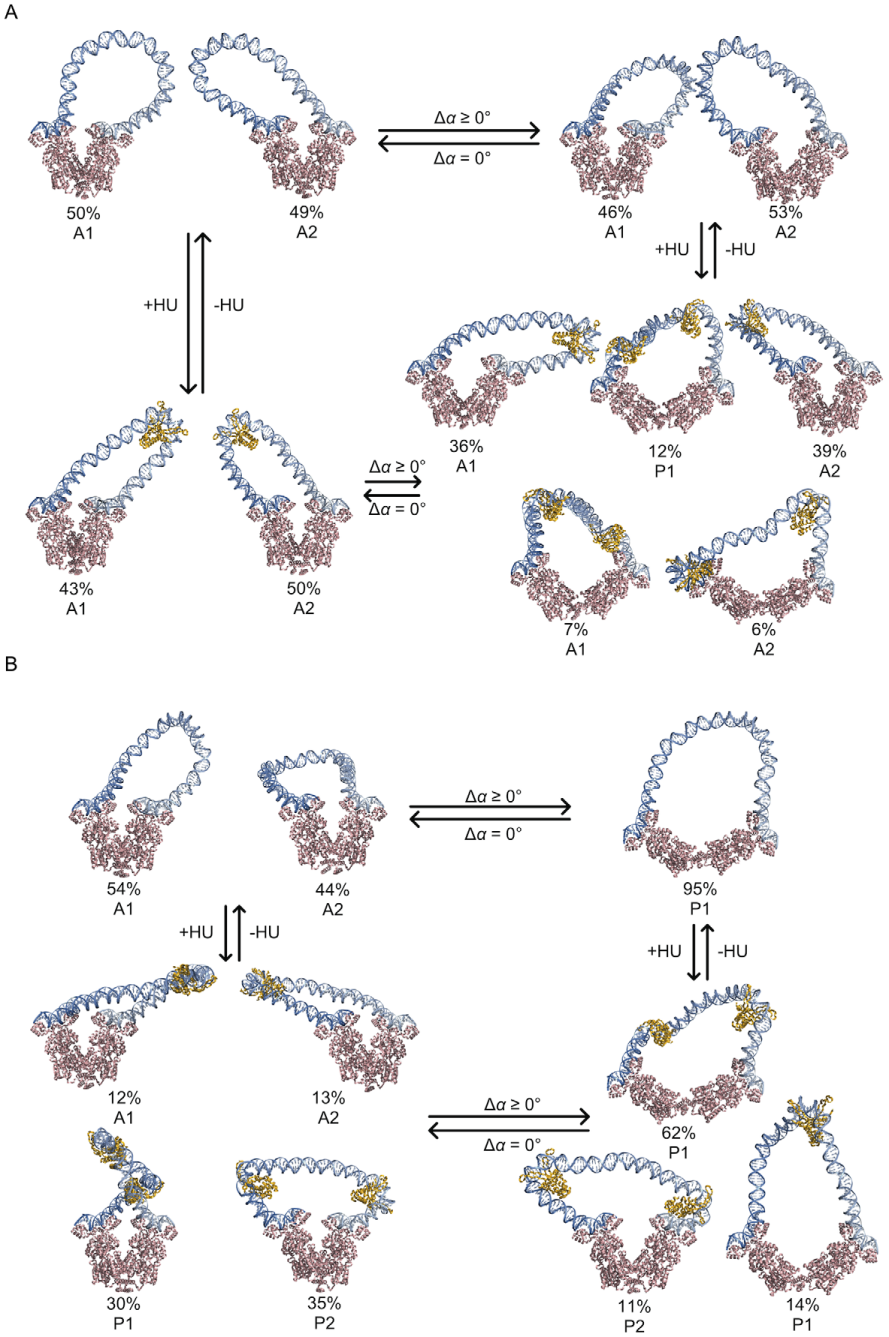
The composite interactions of DNA, LacR, and HU produce a multiplicity of looped states. Molecular snapshots reveal the complex interplay of protein and DNA structure in simulated LacR-mediated DNA with (A) 109 and (B) 115 base pairs between the centers of bound operators. Structures captured from computations performed in the absence and presence of randomly bound HU (upper and lower images in each set) and with allowance for opening of LacR from the V-shaped tetrameric assembly (right and left images in the sets). Images rendered with PyMOL (www.pymol.org) and drawn in a common viewing direction looking down the shortest principal axis of the C^α^ atoms in the LacR assembly, with the core of protein strand A always shown on the left. The DNA is represented by a color-coded backbone (5′ to 3′ chain progression depicted by the dark to light blue color change), and the protein by space-filled atomic representations (LacR in rose, HU in gold). The numbers below the images denote the fraction of loops with the given loop orientation and the illustrated number of bound HU proteins in the simulated ensembles. Addition (+) or removal (−) of HU and prohibition (Δ*α* = 0) or allowance (Δ*α* ≥0) for flexibility in LacR denoted along arrows.

Allowance for opening of LacR preserves general features of the DNA loops formed on the rigid LacR assembly. Specifically, the loops anchored to the deformable template exhibit chain-length-dependent variations in loop type and HU uptake similar to those reported in Figures 3 and 4 for loops attached to the rigid LacR assembly (see Figures S3, S4, S5, S6 in the Supporting Information for histograms and contour surfaces associated with the DNA loops bound to deformable LacR). Loops of the same chain length, however, do not necessarily bind the same number of HU dimers or attach to the LacR headpieces in the same manner. As evident from the molecular snapshots in Figure 7, the opening of the repressor generally perturbs the spatial arrangements of LacR-bound DNA whether or not HU is present. The examples are illustrative of the changes in global structure found in small DNA loops that differ in length by roughly half a double-helical turn and correspond to states near local maxima and minima in the *J*(*N*) profiles. The 109-bp loops, illustrated on the left half of Figure 7A, form with roughly tenfold greater likelihood on the rigid LacR assembly than the 115-bp loops, depicted on the same half of Figure 7B. The corresponding loops formed on the deformable LacR template, shown on the right halves of the two images, occur with equivalent to roughly fourfold greater frequency in the respective absence or presence of HU.

Variation of LacR structure in the absence of HU has limited effect on the configurations of the 109-bp loops, which adopt relatively similar antiparallel arrangements on rigid and deformable LacR assemblies (Figure 7A, top right and left). By contrast, the opening of the protein rearranges the tightly curved, HU-free 115-bp loops anchored in antiparallel orientations on the V-shaped structure to extended forms bound in parallel orientations on the deformable template (Figure 7B, top right and left). The different responses in DNA at the two chain lengths mirror the degree to which the LacR opens upon loop formation. The change in the mean opening angle 〈Δ*α*〉 between the arms of DNA-bound LacR is much smaller for the 109-bp loops for than the 115-bp loops, with respective values of 27° and 87°. The degree of opening in the latter structures is comparable to that found previously to account for the DNase I cutting properties of very short HU-free LacR-mediated DNA loops [27].

The effects of LacR opening on the DNA looping pathways are especially pronounced in the presence of HU. Whereas nearly all the 109-bp loops bound to the rigid LacR protein adopt antiparallel configurations in the presence of HU, those formed on the deformable assembly include a notable proportion of parallel arrangements under the same conditions (Figure 7A, bottom left and right). The fraction of HU-bound 115-bp loops anchored in parallel orientations to deformable LacR exceeds the fraction successfully attached to the rigid LacR template (Figure 7B, bottom right and left). The opening of LacR changes the number of HU dimers bound to the looped DNA. Slightly more HU binds, on average, to loops anchored to a deformable template than to the rigid LacR structure (1.3 versus 1.1 HU dimers per 109-bp loop and 1.9 versus 1.8 dimers per 115-bp loop).

### LacR opening contributes to *in vitro* looping of DNA

**Figure 8:**
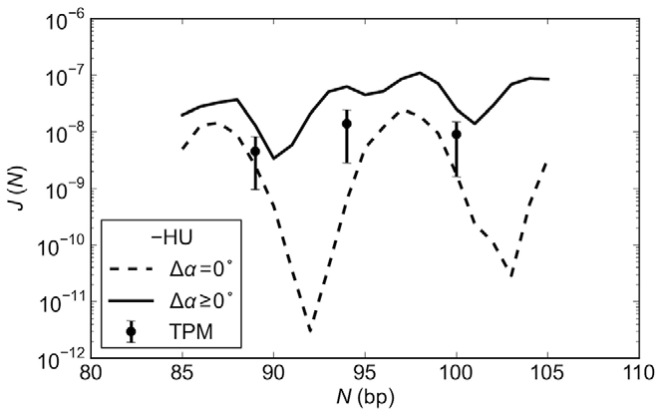
LacR opening contributes to *in-vitro* DNA looping. Comparison of the simulated ease of LacR-mediated DNA loop formation in the absence of HU with the looping propensities deduced from tethered particle motion studies (filled-in circles bracketed by error bars) [36] hints of possible LacR opening *in vitro*. Simulated values are obtained from calculations with a rigid V-shaped protein complex (Δ*α* = 0) and a deformable LacR assembly(Δ*α* ≥0) and connected respectively by dashed and solid lines). See text for discussion of chain extension.

The values of the *J* factors obtained from the simulations performed in the absence of HU bracket the values found for short DNA loops in tethered particle motion studies [36] (Figure 8). The computed likelihood of forming short LacR-mediated loops bound to the headpieces of the rigid tetrameric assembly is slightly lower than the observed measurements, and that of looping DNA on the deformable LacR structure is slightly higher than the experimental data. The latter loops, however, take better account of the observed responses of surface-tethered DNA molecules to internal loop formation than the former. Specifically, the predominant parallel orientations of the HU-free 94-bp loops formed on the deformable LacR assembly, where the increment in the simulated opening angle Δ*α* fluctuates around a mean value of 27°, would have a lesser effect on the end-to-end extension of the long DNAs in which they are embedded than the corresponding 89- and 100-bp loops, which are predicted to adopt antiparallel arrangements on more opened (Δ*α* = 71–79°) LacR structures. Indeed, the DNA chains capable of forming internal LacR-mediated loops of 94 bp in solution are 100–150 Å longer than those with the same O1 and O_sym_ operator sites separated by 89 or 100 bp [36]. The DNA loops attached to the rigid LacR template do not account for this difference in chain extension. Simulated chains of these lengths form on the rigid protein assembly in similar antiparallel orientations.

The predicted occurrences of loops of selected chain lengths anchored in either antiparallel or parallel orientations against opened or closed tetramer structures also provide a rationale for the two distinct types of looping detected experimentally. For example, the predicted configurations of 153- and 158-bp loops appear to account for the motions of beads attached to the ends of constructs with internal loops of these sizes [35]. Whereas the former loops anchor preferentially in antiparallel orientations on partially opened LacR structures, the latter form on both opened and closed tetrameric assemblies (Figure S6 in Supporting Information). The distinct end-to-end differences in the simulated ensembles mirror the tether lengths detected experimentally, *i.e*., a single state for the shorter loops and two states for the longer loops [35].

The opening and closing of the LacR assembly that accompanies the simulated formation of DNA loops of different sizes also accounts for the chain-length-dependent variation in looping topology detected in recent fluorescence resonance energy transfer studies [22]. Notably, both the predicted and observed opening of the tetramer is greater when attached to DNA of lengths less easily closed into loops. Our models, however, do not take account of the long, naturally curved DNA fragments that comprise roughly 60% of the experimentally generated loops and lie in different settings with respect to the *lac* operators. Whereas the naturally straight DNA considered in our computations shows no preference to orient on LacR in one antiparallel form over the other, the centrally positioned curved insert apparently contributes to the propensity of many of the experimental constructs to adopt one antiparallel arrangement in preference to the other. Moreover, differences in DNA sequence near the ends of the constructs, but between the LacR-bound operators, appear to influence the relative populations of loops of the same length. For example, only one of the five 156-bp constructs adopts an extended arrangement consistent with the predicted looping. The other constructs bind to LacR in one or the other antiparallel orientation or sample a mix of antiparallel and parallel states. Interpretation of this rich set of data with a model that takes account of the intrinsic structure and deformability of individual base-pair steps, including the setting of naturally curved inserts, promises to reveal new insights into the sequence-dependent properties of DNA.

## Discussion

### Concerted protein-DNA interactions enhance DNA looping propensities

The stiff, naturally straight double-helical structure of DNA impedes formation of the short, protein-mediated looped structures implicated in bacterial gene regulation. Here we show how perturbations of DNA induced by the random binding of the nonspecific architectural protein HU and deformations of the Lac repressor protein that anchors DNA enhance the likelihood of loop formation. The precise arrangement of the DNA operators on the anchoring protein determines the lengths of duplex most likely to fit between the two binding sites, *i.e*., the number of turns of the double helix with complementary strands in closest register with those on the bound operators. The DNA chain length, in turn, determines the preferred binding orientation of the loops on the protein assembly. Chains with a greater looping propensity on LacR tend to attach to the binding headpieces in antiparallel arrangements and those with a lesser propensity in parallel arrangements. The non-specific binding of the HU dimer introduces sites of localized bending, untwisting, and helical axis dislocation of DNA that not only reduce the separation between points on the chain but also realign the double helix. The changes in local structure appear to contribute to the greater build-up of the architectural protein on the less readily formed DNA loops and the enhanced likelihood of loop formation at such chain lengths. The unanticipated interplay of DNA orientation and HU binding levels (Figures 3 and 4) changes the simple oscillatory pattern of looping preferences of pure DNA into a more complex chain-length-dependent pattern (Figure 2A).

The opening of LacR from the crystallized V-shaped structure similarly changes the strand alignment and three-dimensional spacing of operators. The accompanying changes in the repressor assembly with DNA chain length, however, differ from those associated with HU, so that the combined effects of the two proteins on DNA loop formation are not simply additive. For example, whereas allowance for the binding of HU to loops formed on the rigid LacR template or introduction of opening in LacR in the absence of HU has little, if any, effect on the orientations, shapes, and populations of 109-bp loops, the combined effects of the two proteins generate new and different types of loops (Figure 7A). In other words, the collective behavior of the system is greater than the sum of its parts. Moreover, the composite interactions of proteins and DNA create a multiplicity of looped configurations that bring the predicted looping propensities within range of the levels detected *in vivo*. The structures that account for these data reveal surprising new pathways that DNA may adopt between the headpieces of the Lac repressor.

### The simulated structures capture the observed chain-length dependence of looping

Significantly, the mix of looped states generated in the simulations captures the chain-length dependent oscillations of looping propensities extracted from gene-expression measurements [11]–[13] (Figure 5). The absolute magnitudes of the simulated *J* factors in the presence of HU, however, exceed those associated with the measured repression levels in wild-type cells, and the values of *J* computed in the absence of the architectural protein fall short of the apparent looping behavior of mutant cells that do not express HU. The dampening of the variation in *J* with chain length, brought about by opening of the LacR model, hints of similar global bending deformations in the cellular milieu, albeit with concerted changes in twist consistent with the observed phasing. Consideration of any one of a number of well-known structural features of DNA, HU, or LacR — the sequence-dependent dimeric structure and deformability of DNA [30], the known propensity of HU to bind A+T-rich sequences [37], the subtle differences in the structures of the O_sym_ operators (introduced in our model) *vs*. the O2 operator (used in the cited experiments) on the LacR headpieces [38], [39], *etc*. — could alter the discrepancies in phasing. Indeed, the simple interchange of O1 and O2 operators on similar *lac* constructs shifts the experimentally observed looping propensities such that chain lengths found in one system to be most likely to form loops [11]–[13] become least likely in the other [40] and *vice versa*.

### Multiple protein factors contribute to ‘*in vivo*’ DNA looping

The computations also suggest how both LacR and HU may facilitate the looping of DNA *in vivo*. HU introduces the distortions in DNA needed to form short loops with ease and LacR deformability works in concert with HU to lower the barriers to looping imposed by the DNA twist. The predicted probabilities of loop formation attain the levels observed experimentally only in the presence of HU (Figure 5). The periodic variation in the uptake of HU with chain length (Figures 4, S2, S4) and the concomitant opening and closing of LacR (Figures 6, S5, S6) allow DNA operators with different spacings to align with nearly comparable facility against the tetramer headpieces (Figure 5), albeit via different folding pathways and loop orientations (Figures 3, 7, S3). The correspondence between simulated and measured *J* factors lends support to features of the model that account for these data. For example, until now there was no reason to expect to find different levels of HU on protein-mediated DNA loops of different lengths and, to the best of our knowledge, there have been no efforts to measure these values. The number of bound proteins and the associated populations of parallel and antiparallel loops depend as well upon the assumed binding levels, *i.e*., HU concentration, and the pathways of HU-bound DNA.

Other architectural proteins found in abundance in *E. coli* but not considered in the present calculations may also contribute to the looping associated with *lac* gene repression. For example, Fis, a protein found in even greater abundance than HU during rapid cell growth [41], introduces relatively smooth bends in DNA upon binding [42] and stabilizes DNA looping *in vitro* [43]. The non-specific association of such a protein could potentially account for the underestimates in the looping propensities of mutant HU-free cells in the present work (Figure 5). Conversely, reduction in the assumed levels of HU could bring the predicted *J* factors to the apparent levels found in wild-type cells. A fourfold dilution of HU reduces the simulated cyclization propensities of short DNA chains by the order-of-magnitude difference found here between predicted and observed looping probabilities [6]. Lesser distortions of protein-bound DNA also lower the computed *J* factor. The observed and computed propensities of DNA minicircle formation in the presence of the less severely bent high-mobility-group protein Nhp6A is 1–2 orders of magnitude lower than that found with identical levels of HU depending upon DNA chain length [7]. The *J* factors extracted from experimental data depend upon the assumed concentration of LacR *in vivo*, which in turn reflects uncertainties in both protein number and nucleoid volume (see Methods). The random binding of one HU dimer per 500 bp of DNA brings the computed looping propensities within close range of the behavior of wild-type (WT) cells [44].

### Relationship to earlier ‘parameter-free’ predictions of LacR-mediated looping

The remarkable ease with which short fragments of a naturally stiff DNA molecule can loop between different binding sites on protein assemblies *in vivo* has stimulated a large body of work on the nature of protein-mediated DNA looping, including studies like the present of the Lac repressor-DNA assembly based on the known physical properties of DNA and the constraints imposed by the LacR complex on DNA loop formation. The approach taken here and by several other groups [45]–[51] differs from the frequent practice, mentioned in the Introduction, of fitting a set of model parameters to experimental data and extracting the apparent helical structure and elastic properties of the DNA. There are no free parameters in the model. DNA is described, instead, in terms of known molecular properties, *i.e*., the intrinsic structures and fluctuations of successive base pairs in the free and protein-bound state, and the correspondence with experimental data can be directly related to features of the model.

Our estimates of the looping propensities of DNA anchored to the V-shaped LacR assembly in the absence of HU accordingly overlap with the recent computations of Towles *et al*. [50], who adapted our earlier base-pair-level Monte-Carlo treatment of DNA cyclization [6], [28] to interpret the looping of DNA detected in tethered particle motion experiments. The much looser bounds imposed on the ends of the simulated loops in that work, however, dampen the oscillations in the *J* factor with chain length compared to the variation reported here for corresponding chain lengths. Indeed, the predicted amplitude in *J* is even lower than that found here with allowance for LacR opening. The changes in loop topology associated with HU binding in the present work are similar in spirit to the altered folds of DNA previously reported by us upon incorporation of DNase I and CAP in determination of the minimum-energy configurations of short LacR-mediated loops [27], [29] and by Perkins and associates [48], [49] in the analysis of the loops formed by intrinsically curved DNA sequences. The present calculations and our recent simulations of DNA ring closure [6] are unique in allowing for non-specific placement of HU and likely structural fluctuations in the bound architectural protein. Our strict adherence to anchoring conditions that restrict the DNA operators to the curved pathway found in the crystal complex with the O_sym_ operator [31], however, preclude the formation of loops that wrap on or around the LacR surface. The formation of such loops requires drastic changes in the operator pathways [51]. Incorporation of other motions in the LacR-DNA complex, such as rotations of binding headpieces like those captured in molecular dynamics simulations [46] or deformation of protein-bound DNA operators similar to those detected in NMR measurements [39], may allow for the wrapping of DNA on the LacR assembly and may enhance the likelihood of loop formation beyond the levels found here with hypothetical LacR opening motions. Explicit treatment of electrostatic interactions of the DNA polyanion and the many charged amino acids of LacR and HU may also change the looping propensities. The present calculations introduce no energetic penalties to limit the degree of LacR opening. The allowance for ‘free’ global deformations, however, reveals a chain-length dependence of repressor opening missed in studies that restrict the tetramer to fully closed or extended conformations [50], [52]. The coordinated variation of DNA loop type and repressor opening helps to resolve conflicting interpretation of the *in-vitro* looping properties of DNA, *e.g*., whether the distribution of looped states revealed in tethered-particle motion studies reflects repressor opening or the mix of DNA orientations on a rigid protein. Our findings suggest that the two processes may occur in concert.

### New directions

Consideration of the detailed structures of protein and DNA in simulations of protein-mediated DNA looping reveals new insights into ways that these molecules may interact at a local as well as a global level. The DNA adopts different types of folds at different chain lengths, nonspecific proteins like HU bind in different numbers at these chain lengths, and anchoring proteins like LacR deform to different degrees. The complex interplay of species is surprising and beyond the expectations of conventional, more phenomenological interpretations of DNA looping.

Our direct approach to loop formation allows us to take precise account of the contributions of individual proteins to DNA topology, both at the molecular level presented here and in terms of classic topological variables. We are already taking advantage of rigorous new methods to assess the geometry and topology of the simulated DNA pathways [53] and measuring the changes in the distributions of writhing numbers and total twist of loops generated under different conditions [44]. We can examine the supercoiling of DNA minicircles formed upon cyclization of LacR-looped complexes [17] by combining the simulated structures of loops of different lengths. Superposition of the two types of antiparallel loops formed on a given LacR template, for example, produces a figure 8 configuration. We can also include supercoiling by adding base pairs to the ends of a simulated loop and constraining the linking number of the composite DNA.

We will introduce other molecular species into the simulations as structural information becomes available. For example, there is no currently known structure of the Lac repressor bound to both DNA and isopropyl β-D-1-thiogalactopyranoside (IPTG), a molecular mimic of allolactose that induces transcription of the *lac* operon. Gene expression studies [11] suggest that such a complex may exist since repression persists in the presence of inducer. Moreover, the structure may differ from the LacR complex with DNA alone since the chain-length-dependent expression levels differ in phase. Similarly, accumulating structural information on how the histone-like nucleoid-structuring (H-NS) protein interacts with DNA and brings distant fragments into close contact [54], [55] promises to add new insight into how the protein destabilizes rather than stabilizes small loops [12].

Finally, Monte Carlo simulations of the type employed here offer no insights into the dynamics of looping, such as the rates of loop closure and opening. New methods that take account of time-dependent fluctuations of clamped DNA structures [56] may help in the future to shed light on these processes.

## Methods

### DNA model

DNA is modeled at the level of base-pair steps in terms of six rigid-body parameters: three angular variables (*θ*_1_, *θ*_2_, *θ*_3_) termed tilt, roll, and twist and three variables (*θ*_4_, *θ*_5_, *θ*_6_) called shift, slide, and rise with dimensions of distance [57]. The base pairs are represented by rectangular slabs and the configuration of a DNA segment of *N*+1 base pairs, *i.e*., *N* base-pair steps, is specified by giving, for 1≤*n*≤*N*+1, both the location **r** *_n_* of the center of the slab that represents the *n* ^th^ base pair and a right-handed orthonormal frame 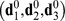 that is embedded in the base pair [58].

The potential governing the fluctuations in base-pair steps is assumed to follow a quadratic expression of the form:
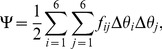
(1)where the Δ*θ_i_* are deviations of the base-pair-step parameters *θ_i_* from their intrinsic values 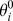 and the *f_ij_* are ‘stiffness’ constants. A configuration of DNA is defined by the set of parameters at each base-pair step and is said to be relaxed when all parameters adopt their intrinsic values. Although the local, sequence-dependent structure and deformability of neighboring base pairs can be incorporated in the 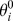 and *f_ij_* [30], the DNA is treated here as an ideal, inextensible, naturally straight molecule with an intrinsic helical repeat of 10.5 bp/turn and the canonical B-DNA rest state and force constants reported in our earlier publications [6], [28]. The allowed bending fluctuations are consistent with the persistence length of mixed-sequence DNA (∼500 Å), the variation in twist is compatible with the equilibrium topoisomer distribution of DNA minicircles [59] and the fluorescence depolarization anisotropy of ethidium bromide molecules intercalated in DNA minicircles [60], and translational parameters are held at the intrisinc B-DNA values (0, 0, 3.4 Å). Use of this simple model, in which all residues are subject to the same types of deformations, is helpful in deciphering the effects of protein on the global properties of DNA.

### Chain construction

Generation of a three-dimensional representation of DNA entails the transformation of the coordinate frame on each base pair into a common reference frame. This is achieved using a serial product of matrices **A** *_n_* that incorporate the 3×1 displacement vector **r** *_n_* and the 3×3 rotation matrix **T** *_n_*_,*n*+1_, which relate the coordinate frames on successive base pairs (*n*, *n*+1):
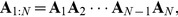
(2)where
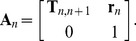
(3)The **T** *_n_*_,*n*+1_ and **r** *_n_* in this expression are generated from the rigid-body parameters at each base-pair step using an analytical formalism [61]–[63] that allows for the characterization of base-pair arrangements independent of chain direction in known structures and the precise reconstruction of models from these values [64], [65]. The **0** is a 1×3 null matrix.

The components of **A**_1:*N*_ yield information about the properties of the chain as a whole. For example, **r**_1:*N*_, the component of **A**_1:*N*_ analogous to **r** *_n_* in **A** *_n_*, is the end-to-end vector of the *N* bp-step chain, and the 3,3 element of **A**_1:*N*_ is the angle *γ* between the normals of terminal base pairs (1 and *N*+1). The trace of the accumulated transformation matrix **T**_1:*N*_ is a function of *γ* and the end-to-end twist *φ* [28].

### Protein-bound DNA

The presence of HU on DNA is modeled, as described previously [6], by incorporation of the appropriate set of base-pair-step parameters in a matrix **A** ^HU^, which is the product of the generator matrices constructed from the rigid-body parameters of the 14 protein-bound steps in one of the four currently available high-resolution complexes of DNA and the HU homodimer from the cyanobacterium *Anabaena* [2] (PDB entries 1P51, 1P71, 1P78). The DNA bound to LacR is similarly expressed by two matrix products, **A** ^O3^ and **A** ^O1^, generated from the rigid-body parameters of the 13 base-pair steps bound to each of the repressor headpieces. In the absence of a high-resolution structure of the LacR-DNA assembly with the O3 operator bound to one half of the complex and the O1 operator to the other, the LacR-bound DNA fragments are assumed to be congruent and are assigned the rigid-body parameters of the palindromic O_sym_ operator associated with the LacR dimer in the 2.6-Å resolution structure [31] (PDB entry 1EFA). The assembly of two such dimers into the V-shaped protein-DNA complex illustrated in Figure 1B is performed by superposition of atoms from the dimer in common with those in each half of the 2.7-Å resolution structure of the tetramer without DNA-binding headpieces [14] (PDB entry 1LBI). The same DNA-binding geometries are used in the opened forms of LacR, which is modeled, as described in more detail below, by rotating the dimeric arms about an axis located within the four-helix polypeptide bundle at the tip of the V-shaped structure and running roughly perpendicular to the plane in which the projection of the tetrameric assembly forms a symmetric V. Motions of this sort appear to connect the known crystalline forms of LacR [14], [15], [21].

The HU, when present on DNA, is placed at random, non-overlapping locations between the LacR-bound operators attached to the ends of the chain. The HU can be positioned with respect to either strand of DNA, yielding eight different HU-DNA configurations for each bound dimer site [6]. The operators are attached to the LacR headpieces in two orientations, *i.e*., pointing toward or away from the symmetry axis passing though the tip of the V-shaped complex and thereby yielding four distinct orientations of the DNA loops on the protein assembly [26], [27] (Figure 1C). The HU binding events introduce an overall bend of 112–129° in the DNA, depending upon the selected template. The cited degree of bending corresponds to the angle between the helical axes of the DNA at the two ends of the protein-bound complexes. The directions of the axes are averages of the local helical axes, computed with the 3DNA software [64], [65], for the last two base-pair steps. The angle between DNA operators depends upon the assumed extent of LacR opening (see below).

### Configurational sampling

Representative configurations of DNA chains are obtained, as described previously [28], by direct Monte-Carlo enumeration using a standard Gaussian random-number generator [66] and a modification of the Alexandrowicz half-chain pairwise-combination technique [67]. The random placement of the nonspecific HU protein on DNA includes corrections for the potential overlap of bound proteins and the generation of partial binding sites on simulated half chains as detailed in full elsewhere [6]. The probability that a site on DNA is occupied by HU, one HU dimer per 150 bp of DNA, corresponds to the level of protein present during the exponential growth phase of *E. coli*, *i.e*., ∼30,000 HU dimers [41] in the presence of a 4.6×10^6^-bp genome [68]. The fixed concentration of HU is consistent with the relatively evenly scattered distribution of HU in the *E. coli* nucleoid [69].

The configurations of DNA chains capable of closing into minicircles or looping between the headpieces of the LacR assembly are identified from the spatial disposition, *i.e*., rigid-body parameters, of terminal base pairs. A joining step, which introduces a phantom base pair at the far end of the chain, is included in the calculations to test for the desired end-to-end placement. The six step parameters that relate the coordinate frames on the first and the last (phantom) base pairs should be null in the wanted configuration. The end-to-end vector **r**_1:*N*+1_, the global bend angle *γ*, and the net twist angle *φ* should also be zero, and the accumulated matrix product **A**_1:*N*+1_ = **A**_1:*N*_ **A** *_N_*_:*N*+1_ thus equal to the 4×4 identity matrix. See Figure S7 in the Supporting Information for a schematic of the constraints placed on the terminal base pairs of a DNA fragment constrained to a specific end-to-end arrangement.

The joining step used in the assessment of cyclization makes use of the Gaussian sampling technique. A randomly configured base-pair step subject to the assumed elastic potential is added through the components of **A** *_N_*_:*N*+1_. The step introduced in the determination of LacR-mediated looping incorporates the rigid-body parameters, found in the complex of LacR with the DNA operators, that relate the coordinate frames on the first and last anchored base pairs, *i.e*., the matrix **T** *_N_*_:*N*+1_ and the vector **r** *_N_*_:*N*+1_ that express the coordinates of the first base pair of O3 and in the frame of the last base pair of O1. The latter parameters depend upon the conformation of LacR and are obtained for the open forms of the protein by relating the aforementioned coordinate frames on O3 and O1 to a coordinate frame embedded in one of the dimeric arms of the V-shaped assembly. The origin of this intermediate frame lies on the α-carbon (C^α^) of Leu342 in the tetramerization domain of the inner protein. The *x*-axis runs from this atom to the C^α^ of Leu71 in the ligand-binding domain of the same protein, and the *z*-axis is perpendicular to the plane that contains the aforementioned atoms and the C^α^ of Thr336 from the tetramerization domain of the second protein in the dimer. The opening of LacR is effected by a rotation Δ*α* about the *y*-axis of this frame. (See Figure S8 and Table S1 in the Supporting Information for molecular images of various degrees of simulated LacR opening and the associated rigid-body parameters of the joining steps used to detect loop closure.) Here, for simplicity, we assume that the opening of the repressor is “free”, with no energy penalty associated with the disruption of the small contact interface believed to stabilize the V-shaped form. Introduction of a penalty term proportional to the surface area of the contact interface in the closed complex does not change the general findings reported herein [44].

Because the chances are very low that the added base pair will superimpose perfectly on the first base pair in any simulated structure, the end conditions are relaxed and only configurations that fall within the following bounds are classified as closed or looped (depending upon the nature of the joining step): (i) **r**_1:*N*+1_<15 Å, (ii) cos *γ* ≥0.98, cos *φ* ≥0.98. The angular limits constrain the trace of **T**_1:*N*+1_ to values very close to 3 and the radial bound limits the distance to excursions no more than 8.8% of the contour length of the shortest (50-bp) sampled DNA chains.

### Gene expression and looping *J* factors

The levels of gene expression determined in wild-type and HU-depleted *E. coli* cells [11]–[13] are converted to *J* factors using the the following method. The measured efficiency of repression is assessed with a quantity called the reporter activity *E*′, defined as the ratio of the raw β-galactosidase activity of a construct with two operators Osym and O2 (and hence capable of forming a loop) and that of a construct with a single operator O2. According to Sadler and Novick [70], these activities are proportional to the probability that the operator closer to the promoter site (the O2 operator in the case of the constructs used in [11]–[13]) is not occupied by a repressor. Following the derivation of Han *et al*. [36], we can evaluate the potential states of binding of a repressor to single-operator and double-operator constructs and express *E*′ as follows:
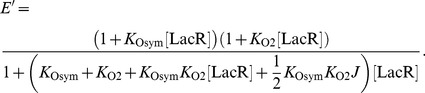
(4)Here *K*_Osym_ is the binding constant of LacR to the Osym operator and *K*_O2_ the binding constant to the O2 operator, for which we take the values *K*_Osym_ = 1.3×10^9^ M^−1^ and *K*_O2_ = 3.6×10^7^ M^−1^. These values are estimated using the method outlined by Zhang *et al*. [24] with the assumed concentration of the LacR tetramer, [LacR] = 103.8 nM, corresponding to the wild-type levels of LacR produced by the engineered plasmid [11]–[13]. The latter value is taken from the known copy number of LacR in *E. coli* detected by equilibrium dialysis (an average of ten tetramers per cell) [71] and the estimated volume of the *E. coli* nucleoid stored in the CyberCell database (0.16 µm^3^) [72]. The concentration is roughly fourfold lower if based on the reported cytoplasmic volume of *E. coli* (0.67 µm^3^) [72]. Equation (4) is then solved for *J* in terms of the measured value of *E*′.

### Molecular reconstructions

Atomic-level representations of the protein-bound DNA loops are generated by concatenation of three molecular fragments: (i) the O3 DNA fragment at the start of the chain and the LacR dimer to which it is bound, (ii) the simulated DNA loop with or without bound HU, and (iii) the O1 DNA fragment at the chain terminus and the associated LacR dimer. The coordinates of the DNA are generated from the base-pair-step parameters using the 3DNA software [64], [65]. The base-pair steps bound to the terminal LacR dimers are assigned the rigid-body parameters found in the high-resolution crystal complex and those on the intervening loop are assigned the parameters found to place the ends of the fragment in the vicinity of the desired arrangement. Any HU molecules bound to the DNA loop are included in the latter set of values. The coordinate transformations that effect the superposition of corresponding DNA atoms from the model and the relevant crystal complex are then used to arrange the protein on DNA. Molecular images are rendered with Chimera [73] or PyMOL (www.pymol.org).

## Supporting Information

Figure S1 **Chain-length dependence of the probability of DNA looping between the headpieces of the rigid, V-shaped LacR assembly in different orientations compared to the likelihood of forming protein-free minicircles of the same chain length.** Note the differences in magnitude and phase associated with the formation of antiparallel (A1, A2) *vs*. parallel (P1, P2) loops and the similar phasing of the *J* factors for the least easily formed (P2) loops and the minicircles, all in the absence of HU. Top to bottom: A1; A2; P1; P2; minicircle.
(TIFF)

Figure S2 **Chain-length dependence of the population of HU molecules bound to the four types of DNA loops mediated by the rigid, V-shaped LacR protein assembly.** Top to bottom: A1; A2; P1; P2. See the legend to Figure 2 for details.
(TIFF)

Figure S3 **Fraction of the four types of DNA loops** ***f*** **_loop_ formed on the deformable LacR assembly and the corresponding chain-length dependence of the** ***J*** **factor determined in (A) the presence or (B) the absence of randomly bound HU molecules.**
(TIFF)

Figure S4 **Fraction of HU molecules** ***f*** **_HU_ bound to DNA loops, of chain length** ***N*** **, mediated by the deformable LacR protein assembly.**
(TIFF)

Figure S5 **Contour plots of looping probabilities, as a function of chain length** ***N*** **and the change in the LacR opening angle Δ** ***α*** **, for loops formed in different orientations on a deformable protein template in the presence of HU.** Distributions are normalized for each plot. See Figure S3 for the relative abundance of each loop type. The blue-to-red scale at the lower right denotes the frequency of loop closure.
(TIFF)

Figure S6 **Contour plots of looping probabilities, as a function of chain length and LacR opening angle, for loops formed in different orientations on a deformable protein template in absence of HU.** See legend to Figure S5.
(TIFF)

Figure S7 **Schematic of the geometric constraints used to determine whether a linear DNA segment meets a specific end-to-end arrangement.** Here a sampled chain of *N* base pairs (green blocks) adopts a configuration that approaches the desired geometry (blue blocks). The end-to-end vector **r** (thick black arrow) joins the *N* ^th^ base pair of the simulated chain to that in the perfectly configured chain. Precise chain alignment requires that the net bend angle *γ* between base normals, the end-to-end Twist *φ* (defined by the long axes and normals of the same base pairs), and the components of **r** are null.
(TIF)

Figure S8 **Molecular images illustrating the opening of the LacR tetramer between the V-shaped model (Δ** ***α*** **= 0°) generated from known crystallographic information and increasingly extended forms (Δ** ***α*** **= 30–120°) incorporated in simulations of DNA looping.**
(TIFF)

Table S1 **Rigid-body parameters (** ***θ_i_*** **,** ***i*** **= 1–6) describing the spatial disposition of DNA operators bound in different orientations on the Lac repressor assembly as a function of the angle of opening, Δ** ***α*** **, between the dimer halves.**
(DOC)
