# Supplementary figures and images for: Interplay of Protein and DNA Structure Revealed in Simulations of the lac Operon
Source: PLoS One. 2013 Feb 14;8(2):e56548. doi: 10.1371/journal.pone.0056548 (PMC3572996; doi:10.1371/journal.pone.0056548)

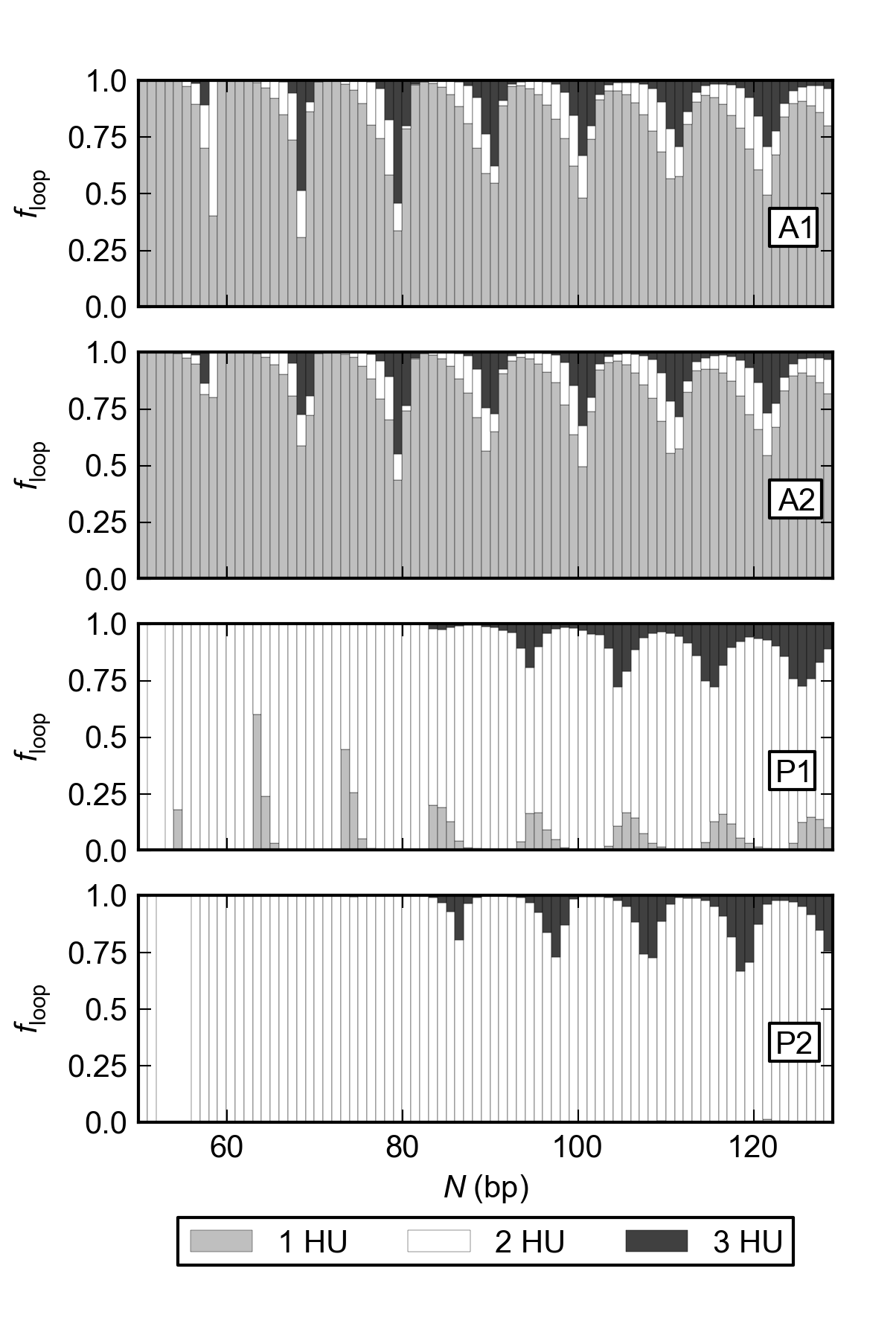

Supplement: Figure S2 — Chain-length dependence of the population of HU molecules bound to the four types of DNA loops mediated by the rigid, V-shaped LacR protein assembly. Top to bottom: A1; A2; P1; P2. See the legend to Figure 2 for details. (TIFF) [file pone.0056548.s002.tiff]

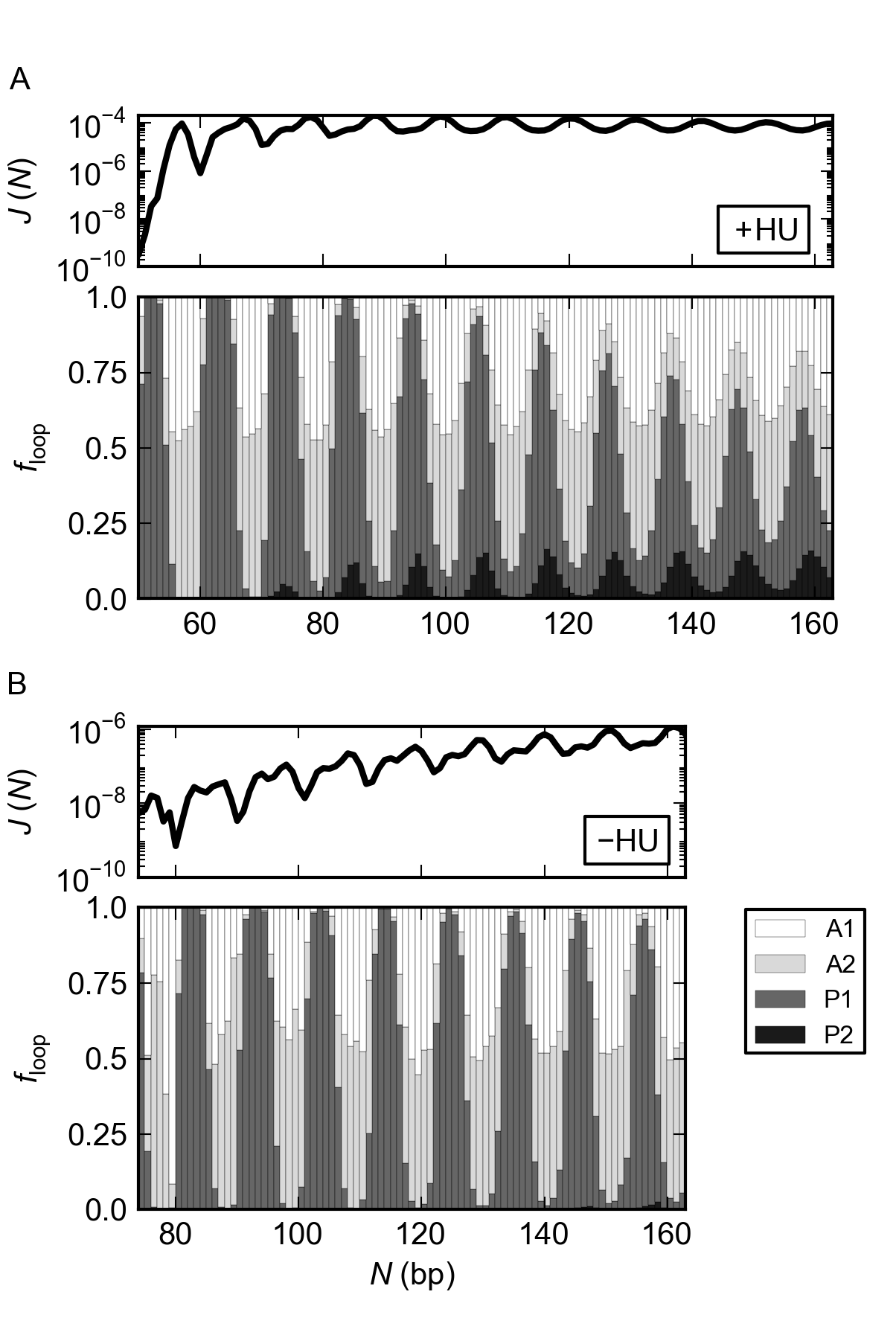

Supplement: Figure S3 — Fraction of the four types of DNA loops f loop formed on the deformable LacR assembly and the corresponding chain-length dependence of the J factor determined in (A) the presence or (B) the absence of randomly bound HU molecules. (TIFF) [file pone.0056548.s003.tiff]

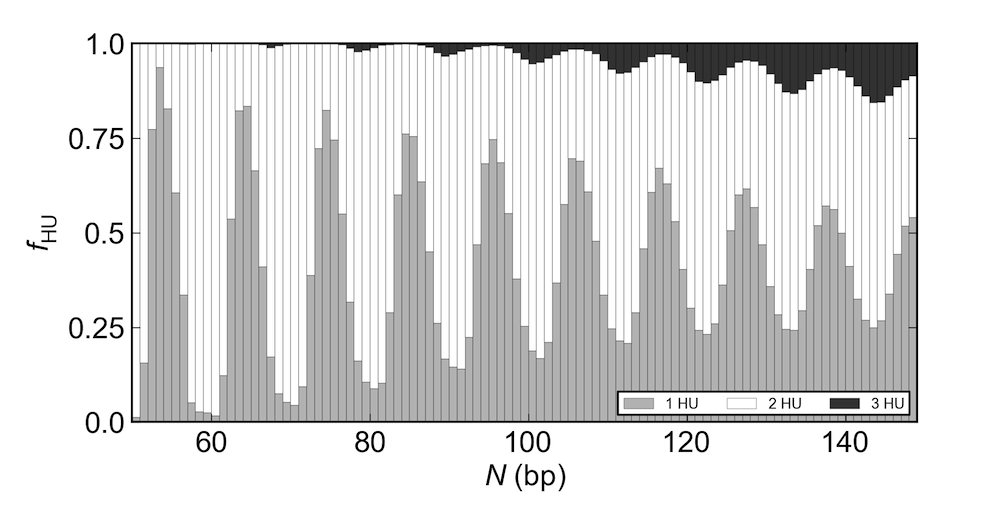

Supplement: Figure S4 — Fraction of HU molecules f HU bound to DNA loops, of chain length N , mediated by the deformable LacR protein assembly. (TIFF) [file pone.0056548.s004.tiff]

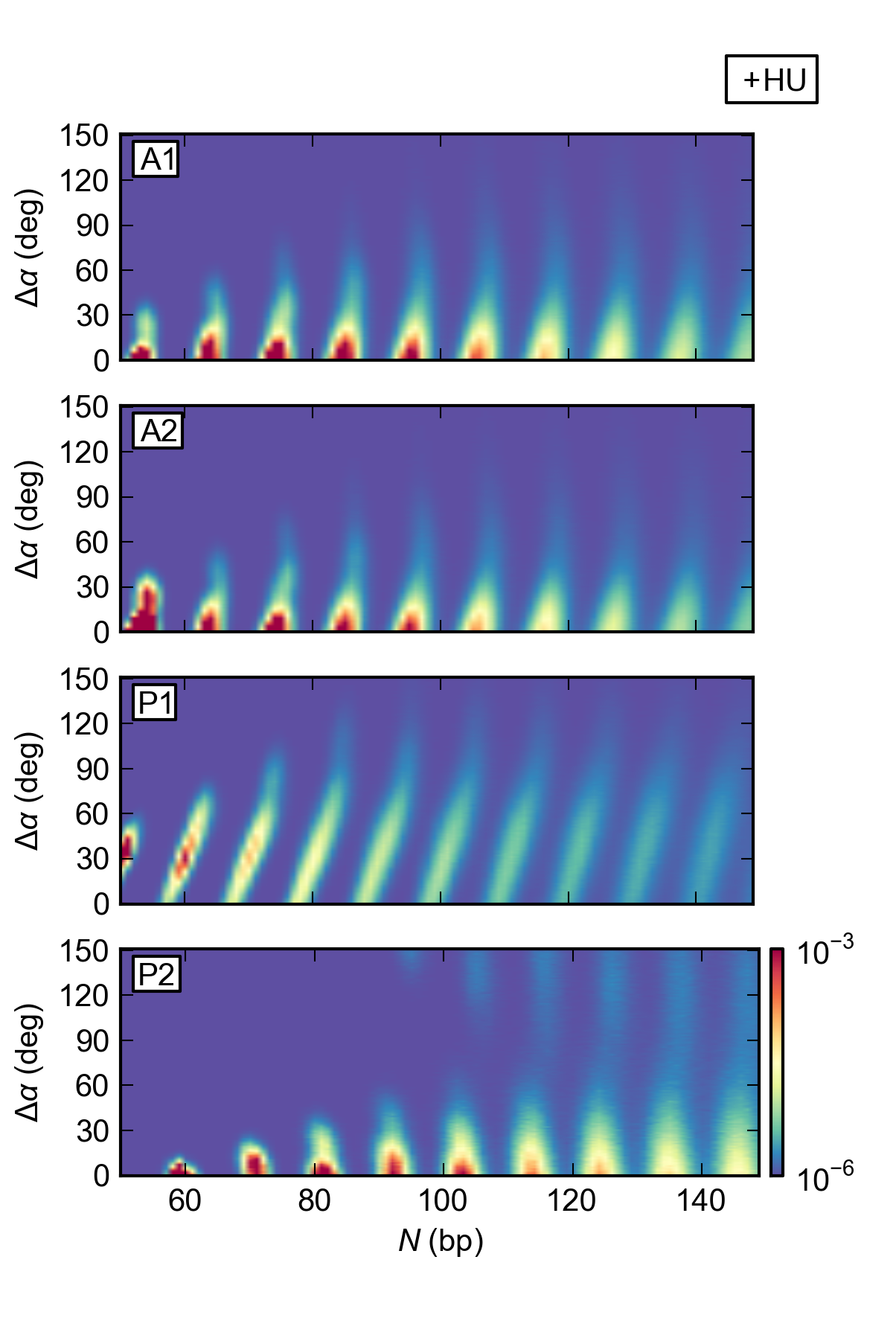

Supplement: Figure S5 — Contour plots of looping probabilities, as a function of chain length N and the change in the LacR opening angle Δ α , for loops formed in different orientations on a deformable protein template in the presence of HU. Distributions are normalized for each plot. See Figure S3 for the relative abundance of each loop type. The blue-to-red scale at the lower right denotes the frequency of loop closure. (TIFF) [file pone.0056548.s005.tiff]

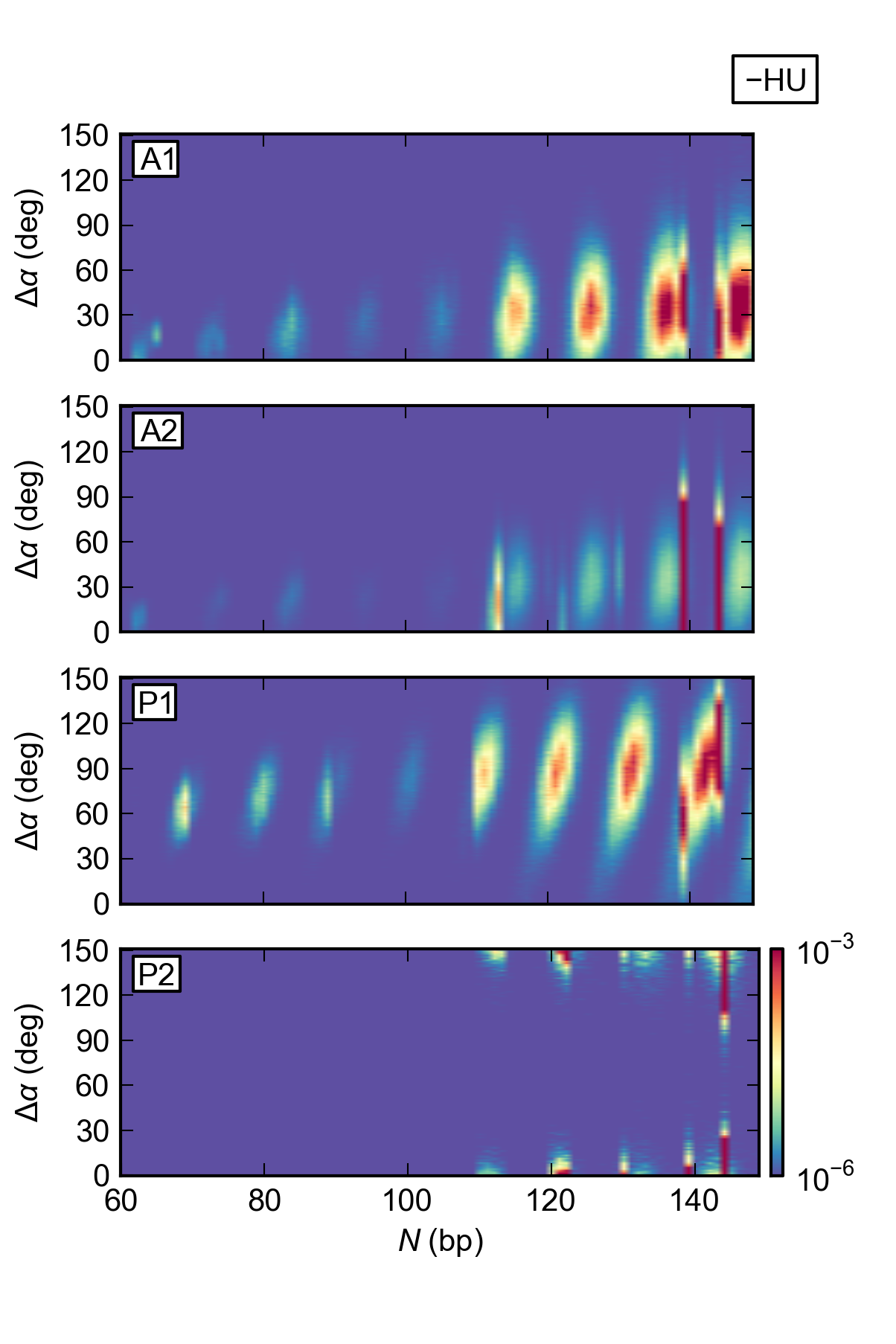

Supplement: Figure S6 — Contour plots of looping probabilities, as a function of chain length and LacR opening angle, for loops formed in different orientations on a deformable protein template in absence of HU. See legend to Figure S5. (TIFF) [file pone.0056548.s006.tiff]

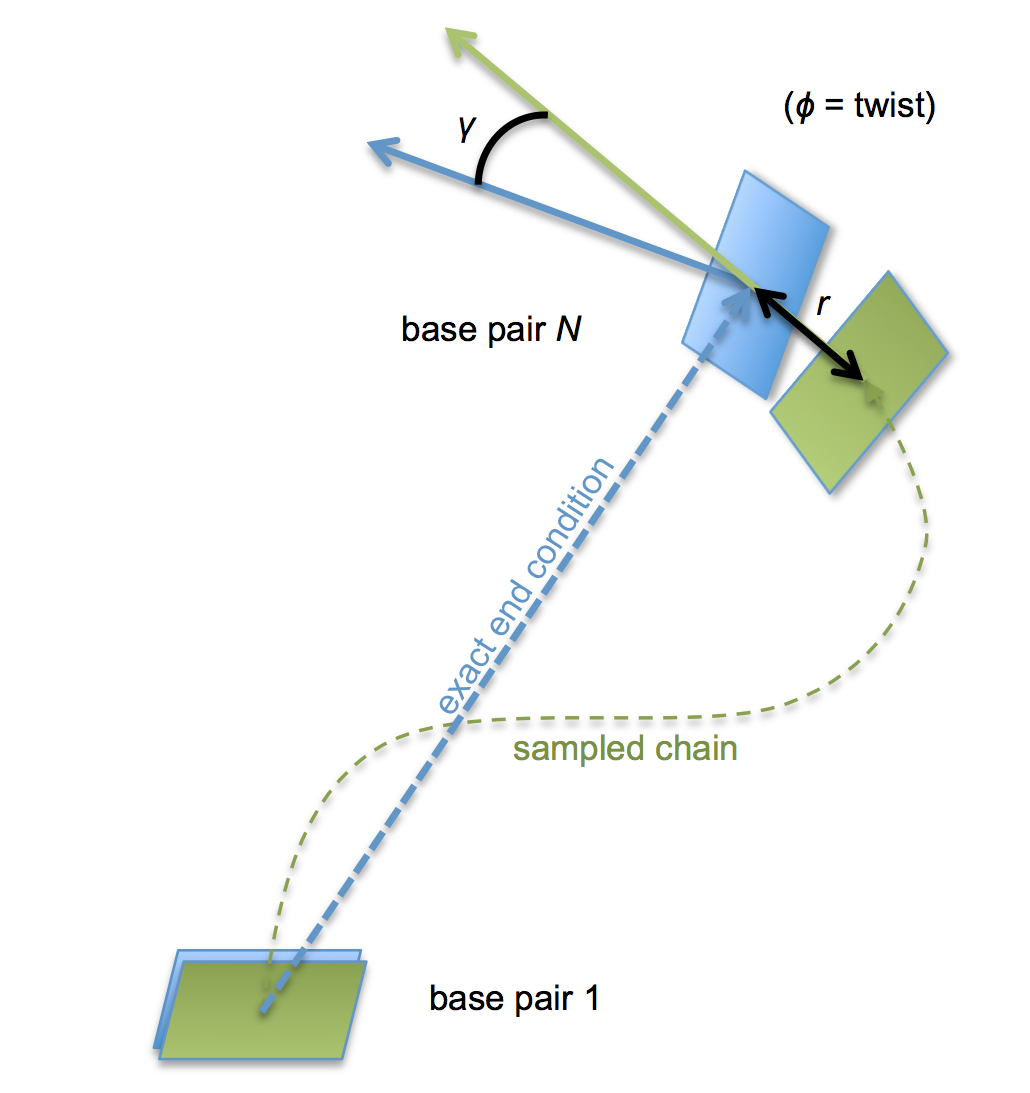

Supplement: Figure S7 — Schematic of the geometric constraints used to determine whether a linear DNA segment meets a specific end-to-end arrangement. Here a sampled chain of N base pairs (green blocks) adopts a configuration that approaches the desired geometry (blue blocks). The end-to-end vector r (thick black arrow) joins the N th base pair of the simulated chain to that in the perfectly configured chain. Precise chain alignment requires that the net bend angle γ between base normals, the end-to-end Twist φ (defined by the long axes and normals of the same base pairs), and the components of r are null. (TIF) [file pone.0056548.s007.tif]

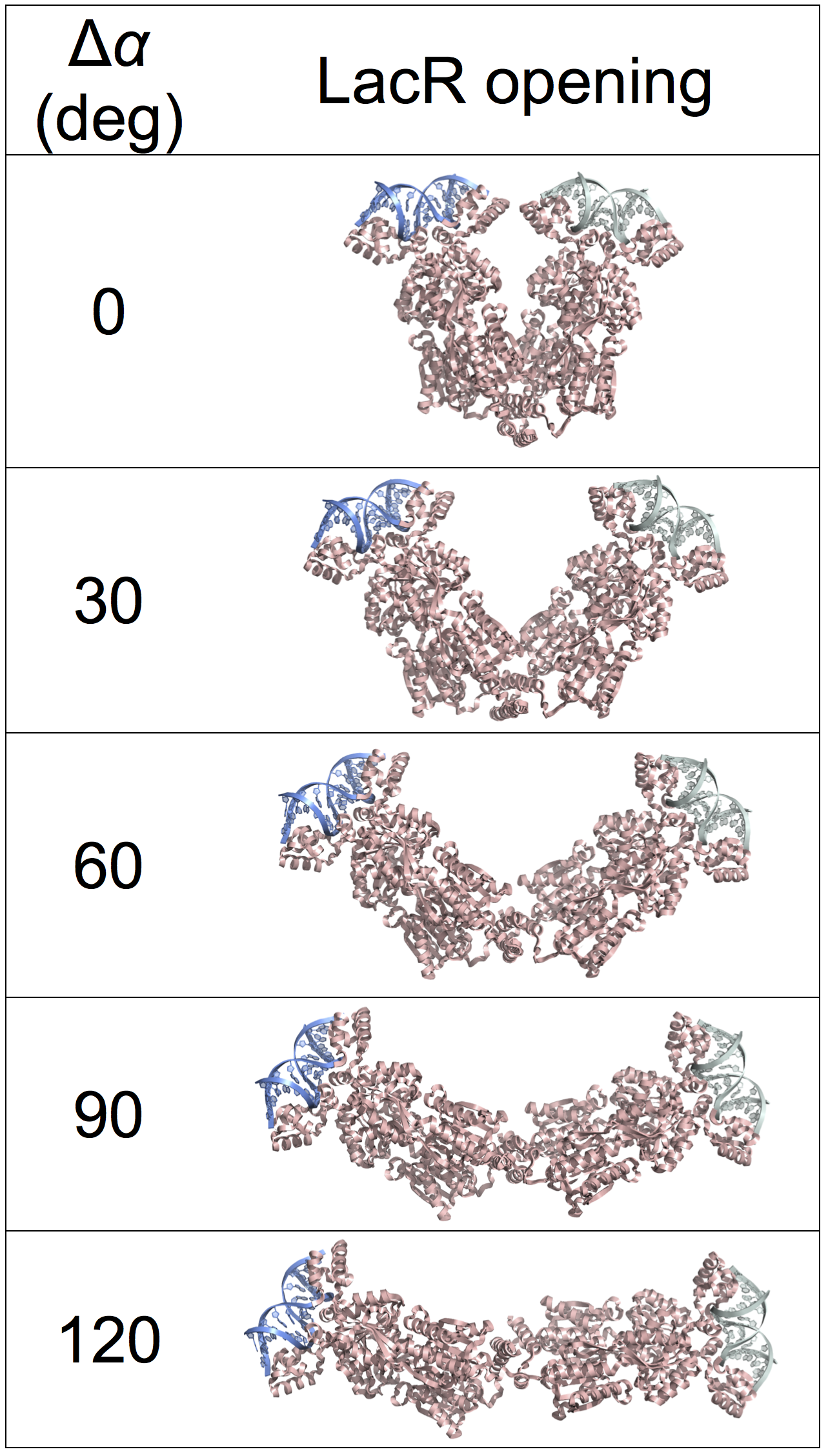

Supplement: Figure S8 — Molecular images illustrating the opening of the LacR tetramer between the V-shaped model (Δ α = 0°) generated from known crystallographic information and increasingly extended forms (Δ α = 30–120°) incorporated in simulations of DNA looping. (TIFF) [file pone.0056548.s008.tiff]
