# Supplementary material for: Interplay of Protein and DNA Structure Revealed in Simulations of the lac Operon
Source: PLoS One. 2013 Feb 14;8(2):e56548. doi: 10.1371/journal.pone.0056548 (PMC3572996; doi:10.1371/journal.pone.0056548)
Supplement: Table S1 — Rigid-body parameters ( θi , i = 1–6) describing the spatial disposition of DNA operators bound in different orientations on the Lac repressor assembly as a function of the angle of opening, Δ α , between the dimer halves. (DOC) [file pone.0056548.s009.doc]

**Table S1. Rigid-body parameters (*θ_i_*, *i*=1-6) describing the spatial disposition of DNA operators bound in different orientations on the Lac repressor assembly as a function of the angle of opening, Δ*α*, between the dimer halves**

|  | Δ*α* (deg) | DNA orientation | Inter-operator step parameters^†^ | | | | | |
| --- | --- | --- | --- | --- | --- | --- | --- | --- |
|  |  |  | *θ*_1_ (deg) | *θ*_2_ (deg) | *θ*_3_ (deg) | *θ*_4_ (Å) | *θ*_5_ (Å) | *θ*_6_ (Å) |
|  | 0° |  |  |  |  |  |  |  |
|  |  | A1 | –148.5 | –38.8 | –41.2 | 18.5 | –31.1 | –67.4 |
|  |  | A2 | 149.5 | –40.4 | –39.4 | –17.8 | –31.4 | –67.0 |
|  |  | P1 | 0.9 | 71.5 | –27.0 | –0.1 | –55.1 | –10.6 |
|  |  | P2 | 0.9 | 56.8 | –118.2 | 0.2 | 44.5 | –99.0 |
|  | 30° |  |  |  |  |  |  |  |
|  |  | A1 | –123.4 | –50.2 | 15.0 | 1.8 | –61.9 | –94.7 |
|  |  | A2 | 123.7 | –51.0 | 16.4 | –3.7 | –57.6 | –98.0 |
|  |  | P1 | 0.5 | 89.6 | 4.6 | –3.4 | –87.0 | 1.0 |
|  |  | P2 | 0.5 | 28.3 | –128.2 | –3.2 | 51.8 | –134.3 |
|  | 60° |  |  |  |  |  |  |  |
|  |  | A1 | –94.0 | –56.6 | 36.7 | –0.7 | –87.0 | –113.9 |
|  |  | A2 | 94.0 | –56.7 | 36.8 | –2.3 | –78.3 | –122.4 |
|  |  | P1 | 0.1 | 101.9 | 45.5 | –6.3 | –118.3 | –12.1 |
|  |  | P2 | 0.1 | –0.7 | –136.9 | –6.6 | 57.8 | –159.3 |
|  | 90° |  |  |  |  |  |  |  |
|  |  | A1 | –63.6 | –59.3 | 48.5 | 2.8 | –104.9 | –126.5 |
|  |  | A2 | 63.5 | –58.8 | 47.8 | –5.6 | –92.5 | –139.8 |
|  | | P1 | –0.4 | 105.4 | 94.0 | –8.8 | –135.8 | –51.7 |
|  |  | P2 | –0.3 | –29.2 | –145.6 | –9.0 | 63.7 | –172.3 |
|  | 120° |  |  |  |  |  |  |  |
|  |  | A1 | –33.0 | –58.9 | 56.6 | 8.6 | –114.5 | –131.5 |
|  |  | A2 | 32.9 | –57.9 | 55.3 | –10.6 | –99.4 | –148.7 |
|  |  | P1 | –0.8 | 99.0 | 140.9 | –10.6 | –129.9 | –98.6 |
|  |  | P2 | –0.8 | –57.7 | –155.6 | –10.9 | 69.8 | –171.7 |

^†^Rigid-body parameters relating the coordinate frame on the first base pair of the LacR-bound O3 operator to the frame on the last base pair of the bound O1 operator, *i.e*., parameters specifying the transformation that expresses the coordinates of base pair 1 of O3 in the frame of base pair 14 of O1.
